# RNA-Seq Can Be Used to Quantify Gene Expression Levels for Use in the GARDskin Assay

**DOI:** 10.3390/toxics14010009

**Published:** 2025-12-20

**Authors:** Robin Gradin, Johan Andersson, Andy Forreryd, Henrik Johansson

**Affiliations:** Senzagen AB, 22381 Lund, Sweden; johan.andersson@senzagen.com (J.A.); andy.forreryd@senzagen.com (A.F.); henrik.johansson@senzagen.com (H.J.)

**Keywords:** skin sensitization, non-animal methods, next generation sequencing, RNA-seq, NanoString

## Abstract

Non-animal methods for identification and characterization of skin sensitizers are continuously evolving, advancing towards more effective, accurate, and informational assays. The GARDskin assay is a scientifically and regulatory recognized assay for the assessment of skin sensitizers. It currently relies on targeted gene expression measurement to derive hazard classifications. With the progression of next generation sequencing technologies, whole transcriptome analysis provides an interesting alternative to the currently implemented targeted gene expression approach. RNA-seq was evaluated for its use in the GARDskin assay as a gene expression quantification method. Based on 24 paired samples acquired on both RNA-seq and the NanoString nCounter platform (the currently standard GARDskin acquisition method), gene expression profiles were found to be highly similar. Comparisons of treatment effects yielded a Spearman’s correlation coefficient of 0.95 and a Lin’s concordance correlation coefficient of 0.87. RNA-seq data was also used to classify the sensitizing hazard of 24 treatments using the standard GARDskin analysis pipeline. The classifications corresponded completely with references, rendering correct classifications for all treatments. In conclusion, it was found that the RNA-seq data strongly resembled NanoString nCounter data, and that it could be used to derive reliable hazard classifications in the GARDskin assay.

## 1. Introduction

Allergic contact dermatitis (ACD) is caused by small molecular weight chemicals that are able to penetrate the protective skin barrier, bind to endogenous proteins while inducing an inflammatory milieu via, for example, triggering of innate danger signals, thereby initiating an immune response that results in the formation of a specific immunological memory towards the chemical [1,2,3]. Repeated exposure to the same chemical elicits the immune response that gives rise to the adverse effects typically associated with ACD.

While there are interindividual and environmental factors that affect both the susceptibility and severity of ACD [4], the underlying pathomechanism of the condition is relatively well established [5], which has facilitated the development of several assays for identification and characterization of skin sensitizers. The earliest such assays were the in vivo variants [6,7,8,9,10,11], which are progressively being replaced by non-animal alternatives, which include in chemico [12,13,14], in vitro [15,16,17,18,19,20,21], and in silico [22] approaches. Non-animal methods is a continuously evolving field, and new methods and improvements to existing assays are being actively developed and presented [23,24,25,26,27,28,29].

The Genomic Allergen Rapid Detection (GARD) assay for assessment of skin sensitizers (GARDskin) is one such in vitro test method. It is based on a human dendritic cell-like cell line and was developed for hazard identification of skin sensitizers [16,30,31]. It is described in the OECD test guideline 442E [32] as a method for monitoring dendritic cell activation, which is the third key event (KE) of the adverse outcome pathway (AOP) for skin sensitization [2]. The first developmental steps towards the formalization of the GARDskin assay were described by Johansson et al., 2011 [16], where whole transcriptome analyses of a dendritic cell-like cell line treated with a panel of established skin sensitizers and non-sensitizers were used to identify a predictive subset of genes capable of effectively separating the sensitizing chemicals from the non-sensitizing ones. The identified gene set included genes that could be linked to biologically meaningful cellular processes, including dendritic cell activation and maturation, and cytoprotective pathways. The wider biological effects of chemical sensitizers in the GARDskin cell system have been examined in detail elsewhere [33]. Following the initial discovery study, the GARDskin assay has been standardized [30], validated [31], shown to be effective on chemical subsets typically considered difficult to test [34,35,36], and further extended to enable medical device classifications [37] and potency predictions [23,38]. It predicts hazard outcomes from the induced gene expression patterns in the genomic prediction signature, termed the GARDskin prediction signature (GPS), using a trained and fixed support vector machine (SVM) (as detailed in the supporting document to the GARDskin assay [39]). The gene expression quantification is performed using a targeted acquisition approach, relying on the NanoString nCounter platform. While the assay was originally developed using Affymetrix arrays, standardization requirements prompted the transition towards a simpler quantification platform, as elaborated on by Forreryd et al. [40], which resulted in a transfer to the NanoString platform [30].

The NanoString nCounter platform has several properties that are advantageous for standardized quantification of defined gene sets. For example, the platform allows for simultaneous quantification of up to 800 target genes; the experimental protocols are simple and easy to implement and standardize; the required hands-on time is low, and the time from experimental initiation to data availability is short; the quantified data is simple to handle and analyze as the targeted transcripts are automatically quantified by the platform without need for further processing. Finally, the platform’s readouts have been shown to be robust and reproducible [41].

Despite this, it is still relevant to explore other gene expression quantification platforms that may be compatible with the GARDskin assay, which is an endeavor that has also been performed previously for other biomarker-based assays [42,43]. By, for example, extending the number of compatible acquisition platforms, the assay may become more accessible. One alternative technology capable of producing quantified gene expression measurements is next generation sequencing (NGS), particularly RNA sequencing (RNA-seq), which has a wide user base and is implemented in many laboratories worldwide [44]. It has matured into a robust acquisition technology over the last decades, and experimental components are available from several different providers [45,46]. While generated data still require significantly more processing before gene level abundance estimates are obtained compared with the NanoString platform, many available tools have been designed with both efficiency and accuracy in mind [47,48,49,50]. In addition, commonly used peer-reviewed pipelines have also been packaged into user-friendly interfaces which reduces the potential hurdle of handling and generating reproducible NGS data [51,52]. Finally, while a targeted quantification approach is effective when genomic targets are known, whole transcriptome data is a technically feasible alternative [53], with one of its main benefits being that the acquired data is not limited to a priori defined gene sets, which significantly extends the ability to perform secondary analyses, including exploratory analyses and differential expression testing [54]. In the context of standardized testing, these types of secondary analyses may be used to enhance the understanding of potential perturbances’ cellular effects.

Therefore, the main objective of this study was to investigate if NGS technology could be used as a method for quantifying gene expression data in the GARDskin assay. In this work, comparisons of NanoString nCounter and RNA-seq data from paired samples show that the genes in the GPS can be reproducibly quantified on both platforms. Notably, the sequencing experiments were performed by an independent laboratory with encoded sample identities. The study also showed that data quantified with RNA-seq can be efficiently used as a data source in the existing GARDskin analysis pipeline to produce accurate hazard classifications.

## 2. Materials and Methods

### 2.1. Overview of the Study Design

The main objective of the study was to evaluate if GARDskin classifications could be accurately obtained from gene expression data quantified using RNA-seq, as an alternative quantification method to the NanoString nCounter platform (Bruker Corporation, Billerica, MA, USA) described in the GARDskin protocols [31,55]. To this end, the study was set up in two main parts.

The first part aimed at comparing gene expression data between the two platforms using paired RNA samples. A total of 24 paired RNA samples were acquired on both platforms. These corresponded to 12 replicates of the GARDskin unstimulated control and 12 replicates of the GARDskin positive control p-phenylenediamine (PPD). These samples were collected to allow for direct comparisons across platforms, and to optimize the reconstruction of NanoString probe signals on the RNA-seq platform. The paired RNA samples were collected from experiments consistent with the GARDskin protocols [31,55], and did not rely on reference RNA such as the Universal Human Reference RNA (UHRR).

The second major part of the study included 75 additional RNA samples acquired on the RNA-seq platform only. These samples corresponded to 22 different chemical treatments and the GARDskin positive and unstimulated control. The main purpose of these samples was to assess the predictive capacity of gene expression measurements quantified with RNA-seq.

To further strengthen the integrity of the study and of any results obtained, the RNA-seq analysis was performed by an independent contract research organization (CRO). The CRO obtained all the RNA samples encoded, and the sequencing experiments were performed without disclosure of sample identities.

### 2.2. Chemicals for Exposure Experiments

All chemicals selected for the evaluation of the NGS platform are described in Table 1. All chemicals but 2-nitro-p-phenylenediamine, propyl gallate, and chlorobenzene were purchased from Sigma-Aldrich (MilliporeSigma, Burlington, MA, USA). 2-Nitro-p-phenylenediamine, propyl gallate, and chlorobenzene were purchased from Fisher Scientific (Thermo Fisher Scientific, Waltham, MA, USA). The chemicals included all of GARDskin’s proficiency chemicals (defined in TG442E [32]). The complete dataset was chosen to ensure that a wide potency range was included while also ensuring that non-sensitizers were present. Furthermore, all chemicals were described in annex 2 of guideline 497 [56] and had previously been correctly classified by the GARDskin assay [30,31,57]. The human and LLNA references, including LLNA Median-Like Location Parameter (MLLP) EC3 values and binary calls, were extracted from annex 2 of guideline 497 [56] (where human calls were based on the human predictive patch tests databased described by Strickland et al., 2023 [58]) for all chemicals except chlorobenzene. The LLNA reference for chlorobenzene was obtained from Gerberick et al., 2005 [59] and from the European Chemicals Agency (ECHA) registration dossier (https://echa.europa.eu/substance-information/-/substanceinfo/100.003.299, accessed on 21 October 2025). A consensus reference call was established for every chemical by coalescing human and LLNA data, prioritizing human evidence if the references were different.

Thus, the overall dataset included both sensitizers and non-sensitizers. The sensitizers included chemicals of varying potencies, ranging from very weak to extreme. Furthermore, the selected chemicals included a range of reaction chemistries, including reactivity domain alerts for SN2, SNAr, Schiff base formations, and Michael acceptors [60].

### 2.3. Cell Exposure Experiments and Total RNA Isolation

All exposure experiments were conducted in compliance with the standard GARDskin assay protocols [55].

The SenzaCell cell line (ATCC Depository PTA-123875) was exposed to each of the chemicals at a concentration that induced 85–95% relative viability while not exceeding a maximum exposure concentration of 500 µM. Chemicals that did not affect the cells’ viability were run at a maximum of 500 µM or the highest soluble concentration. All treatment concentrations are described in Table 1. Cells were incubated with the chemicals for 24 h, after which relative viabilities were reconfirmed, and cells were harvested and lysed in TRIzol (Thermo Fisher Scientific, Waltham, MA, USA), before total RNA was isolated. The quality and the concentration of the isolated total RNA were determined as described below (see the quantification sections) before being used as input into the respective quantification pipelines. The viability measurements were performed using propidium iodide staining and flow cytometry analysis.

### 2.4. NanoString nCounter Quantification

The NanoString quantification was performed in accordance with the provider’s protocols, as detailed in the GARDskin assay protocols [55]. The quality and the concentration of the RNA samples were determined using Agilent Bioanalyzer 2100 (Agilent Technologies, Santa Clara, CA, USA).

### 2.5. RNA Sequencing

Total RNA quality was evaluated using Agilent TapeStation 4200 (Agilent Technologies, Santa Clara, CA, USA), and the RNA concentration was estimated using Qubit Flex (Thermo Fisher Scientific, Waltham, MA, USA). The library was prepared using Illumina^®^ Stranded mRNA Prep, Ligation (20040534, Illumina, San Diego, CA, USA) per the provider’s protocols (Illumina Stranded mRNA Prep, Ligation Reference Guide [Document # 1000000124518 v03]). The library’s quality and concentration were assessed using Agilent TapeStation 4200 and Qubit Flex, respectively. Sequencing was performed on a NovaSeq 6000 (Illumina, San Diego, CA, USA), generating paired-end reads of 150 bp, using NovaSeq S2 Reagent Kit (Illumina, San Diego, CA, USA), 300 cycles v1.5.

### 2.6. Quantification of RNA-Seq Data

The quality of the sequenced data was assessed using fastQC version 0.12.1 (https://www.bioinformatics.babraham.ac.uk/projects/fastqc/ (accessed on 13 October 2025)). Reads were trimmed for adapter sequences and low-quality calls using fastp version 0.23.4 [49]. Transcripts were quantified using salmon version 1.10.0 [48] with selective alignment enabled [50], using the entire genome as background. The quantification was made against an index constructed from human GENCODE reference, release 47 (GRCh38.p14) [61]. The complete parameter set given to the respective program is detailed in Supplementary Table S1. No pre-defined exclusion criteria were set for the identification of outlier samples. The quality control revealed no samples of questionable quality, and all samples were retained and included in the analysis.

### 2.7. Mapping Candidate Transcripts to the NanoString GARDskin Prediction Signature

In order to reconstruct the NanoString signal on quantified RNA-seq data, transcripts that were likely targets of the NanoString probes were first identified. Target transcripts were identified by comparing the reference transcriptome’s sequences (GENCODE release 47) to the 100 bp NanoString probes’ binding sequences [39]. The alignments between probe sequences and the reference transcriptome were made using Bowtie 2 version 2.5.4 [62]. NanoString probes that did not obtain any mapped reference transcript were assigned all transcripts that were annotated to the NanoString probe’s target gene. Following this step, each of the 196 NanoString probes had been assigned at least one candidate transcript available for quantification using RNA-seq.

### 2.8. Refining the RNA-Seq Candidate Transcripts for NanoString Signal Reconstruction

The sets of candidate transcripts identified via sequence alignments were optimized to maximize the resemblance between the two platforms’ signals. The optimization was performed using a backward elimination procedure relying on the 24 paired samples acquired on both platforms (see above).

Firstly, for the optimization, the RNA-seq transcript abundance estimates were expressed in transcripts per million (TPM), as obtained from the salmon quantification step. TPMs were used as it corrects for both library size and for gene lengths. Similarly, individual NanoString samples were normalized using a scaling factor based on the total number of acquired counts [30], as described in Equation (1). This normalization was chosen as it constitutes the first step in the standard GARDskin analysis pipeline, aiming to reduce for variation in library size [30].

(1) $$y_{ij}=\frac{{count}_{ij}}{\sum_{j=1}^{n}{count}_{ij}}$$

where *y_ij_* is the normalized expression value for gene *j* in sample *i*, and *count_ij_* is the observed count for gene *j* in sample *i*.

Then, each of the 196 genes were processed independently, with the aim of minimizing the difference between NanoString data for an individual probe with the reconstructed signal from RNA-seq. A reconstructed RNA-seq signal was obtained by adding all remaining candidate transcripts’ abundance estimates for a particular sample. The difference between the two platforms were calculated using the mean absolute error, as expressed in Equation (2), of log-transformed and centered abundance estimates (see Equation (2)).

(2) $$d_{j}=\frac{\sum_{i=1}^{n=24}\left|\left(\log_{2}y_{ij}-\frac{1}{N}\sum_{k=1}^{N}\log_{2}y_{kj})-(\log_{2}z_{ij}-\frac{1}{N}\sum_{k=1}^{N}\log_{2}z_{kj}\right)\right|}{24}$$

where *d_j_* is the difference for gene *j* between the platforms, *y_ij_* is the normalized expression value for sample *i* of gene *j* quantified on the NanoString nCounter platform, and *z_ij_* is the reconstructed RNA-seq signal for gene *j* and sample *i*.

For the backward elimination procedure, each of the remaining transcript candidates were held out from the signal reconstruction and the difference was recorded. The transcript whose exclusion produced the lowest difference compared to the NanoString data was excluded. This approach was repeated until only one transcript remained. The final set of transcripts used to reconstruct a NanoString probe’s signal was the set of transcripts that produced the overall lowest difference between the platforms.

### 2.9. Comparison of Expression Levels Between the Platforms

Having defined the transcript sets to reconstruct the GPS on RNA-seq data, the gene expression levels were compared between the platforms. To this end, the data from both platforms were first normalized using a similar strategy, based on the 196 quantified genes. The normalization was a simple scaling strategy, based on size factors calculated for each of the datasets independently.

The normalization was performed as follows: firstly, based on the 24 samples in each dataset, an average expression level was determined for every gene by calculating the geometric mean of its expression across samples (see Equation (3)).

(3) $$\mu_{j}=e^{\frac{1}{N}\sum_{i=1}^{N}\log y_{ij}}$$

where *μ_j_* is the average expression for gene *j*, *N* is the number of samples (*N* = 24), and *y_ij_* is the abundance estimate for sample *i* and gene *j*. For NanoString, the abundance estimate was the original counts, while it was the reconstructed probe signals based on TPMs for the RNA-seq data.

A scaling factor was then calculated for each sample by comparing its individual expression levels with the dataset’s average. The comparison was made based on a robust geometric mean of ratios. Compared with the standard geometric mean, the Huber M-estimator of location [63] was used to determine the average difference for the individual samples. Equation (4) shows the derivation of the individual samples’ scaling factors.

(4) $${sf}_{i}=e^{f\left(\log\frac{y_{ij}}{\mu_{j}}\right)}$$

where *sf_i_* is the scaling factor for sample *i*, *y_ij_* the expression level for sample *i* and gene *j*, *μ_j_* the average expression level for gene *j* as determined in Equation (3), and *f* the calculation of Huber M-estimate of location, as implemented in the R-package DescTools version 0.99.47 [64].

Each sample was finally divided by the calculated scaling factor to obtain normalized expression levels for subsequent comparisons. To avoid issues with zeros, all NanoString counts less than an estimate of the limit of detection (LOD) were replaced by the value of the LOD. The LOD for the NanoString platform was estimated as the average across the samples’ median negative control probe counts (LOD = 14). The same strategy was also used for the RNA-seq data, where the LOD was set to a pseudocount of five.

The normalized abundance estimates were compared between the platforms by calculating the geometric average of each gene using the paired replicates of the unstimulated controls (*N* = 12) for both platforms. The association between the platforms’ estimates was examined visually (scatter plots) and by calculating both linear (Pearson) and non-linear (Spearman’s) correlation coefficients. Deviating abundance estimates between the platforms were identified using standard scores based on the log-transformed ratios between the estimates. The standardization was achieved using robust estimates of location (Huber M-estimator of location) [63] and scale (Qn scale estimator) [65], as implemented in the R-packages DescTools version 0.99.47 [64] and the robustbase version 0.99-0 [66].

The comparison of the effect estimates by the positive control treatment across the platforms was performed using linear regression on log-transformed normalized data. The main term of interest was the interaction effect between treatment and platform. Significantly deviating genes were identified as those with a *p*-value adjusted for multiple hypothesis testing [67] below 0.05 and a fold change difference of at least 1.5. In addition to these statistics, the association of the effect estimates was characterized by Spearman’s correlation. Furthermore, Lin’s concordance correlation coefficient was calculated as the association and was expected to be the same independent of the platform.

### 2.10. Generation of GARDskin Predictions

GARDskin predictions were obtained for the 75 samples acquired for assessment of predictive capacity with the complete standard GARDskin analysis [39,55]. The RNA-seq data input comprised the reconstructed signals as described above, which were fed into the standard GARDskin analysis pipeline. The pipeline includes the following steps: normalization using counts per total counts (CPTC; Equation (1)) based on the 196 genes, BARA normalization to reduce differences between batches [39,68], and classification using the already trained SVM. Complete details, including fitted parameter values are described in the Supporting Document to the Genomic Allergen Rapid Detection Test Method for Skin Sensitisation (GARD^TM^skin), described in Test Guideline 442E [39].

The GARDskin analysis pipeline has been developed and defined in a series of publications [16,30,31]. The 196 genes were identified in a data-driven manner by optimizing the discriminatory ability, characterized by the Kullback–Leibler divergence, of an SVM [16]. The CPTC normalization was discussed and described in Forreryd et al., 2016 [30] as a method for reducing technical variance associated with RNA content and quantification levels (library size). The BARA normalization was implemented to remove batch effects between the GARDskin training set that was used to fit the classification model (SVM) and every subsequently acquired test set. Finally, the classification model is a linear SVM trained on a dataset of 38 chemicals, of which 20 were known non-sensitizers and 18 were well-characterized skin sensitizers [30]. The entire pipeline has undergone extensive validation, including testing of previously unseen test chemicals, a ring-trial study [31], and an independent peer review by the Joint Research Center’s EU Reference Laboratory for alternatives to animal testing (EURL ECVAM) (Scientific Advisory Committee (ESAC)) [69].

### 2.11. Assessment of Sequencing Depth on GARDskin Classifications

The impact of the sequencing depth on GARDskin classifications was examined using two approaches. The first less computationally expensive method was binomial subsampling on the already quantified expression matrix. The quantified expression matrix was subsampled at levels between 50,000 and 25,000,000 counts. The subsampling was repeated 100 times for every level. The reduced data was fed through the standard GARDskin analysis pipeline as described previously to generate predicted decision values.

The second more computationally expensive approach was to apply the subsampling to the reads in the fastq files. The quantification and prediction pipelines were then performed as already described. Two levels of subsampling were performed and an average of five or ten million reads were retained. The subsampling was performed using fq version 0.12.0 (https://github.com/stjude-rust-labs/fq (accessed on 13 October 2025)). See Supplementary Table S1 for the detailed function call.

The performance of the subsampling was evaluated with respect to the likeness of the predictions obtained on the full dataset, and with respect to the overall classification performance. The deviation from the predictions on the complete dataset was quantified using the root mean squared error (RMSE) to compare decision values, and the prediction performance with respect to reference labels was evaluated using binary cross-entropy. Decision values were transformed with the standard logistic function before the binary cross-entropy was calculated.

### 2.12. Visualizations

All visualizations were created using R version 4.2.0 [70] and the ggplot2 package version 3.5.2 [71].

The principal components of the RNA-seq data were visualized by aggregating technical replicates using limma version 3.52.0 [72] prior to the singular value decomposition. Aggregated data on log-scale was centered and standardized before the principal components were calculated.

## 3. Results

### 3.1. Reconstructing the NanoString Probe Signals from RNA-Seq Data

The GARDskin assay is an existing, scientifically reviewed and validated [30,31], and regulatory-approved [32] assay for hazard assessment of skin sensitizers. The current state of the assay, as described in the OECD TG442E, uses an analysis pipeline and prediction model defined and trained on gene expression data originating from the NanoString nCounter platform. The prospect of using RNA-seq data in the GARDskin assay requires that the assay’s currently defined models remain unaltered and that cross-platform predictions can be accurately made. To evaluate this, RNA-seq data were acquired for 24 different treatments (see Table 1). Paired samples (acquired using both RNA-seq and NanoString) were acquired for the positive control (p-Phenylenediamine; *N* = 12) and the unstimulated control (*N* = 12).

The RNA-seq data were first adapted for compatibility with the existing GARDskin model’s components by attempting to reconstruct the signals of the 196 measured genes of the NanoString platform. The target sequences of the NanoString platform were mapped to the transcriptome’s FASTA sequences to identify candidate transcripts for signal reconstruction. All but one of the NanoString target sequences could be mapped to at least one reference transcript. The only unmapped target sequence, corresponding to the gene *COX20*, was assigned all reference transcripts annotated as belonging to the gene *COX20*. The sets of candidate transcripts were refined by comparing reconstructed signals with NanoString data for the paired samples, applying a backward elimination procedure to eliminate transcripts from the respective set to maximize the likeness between RNA-seq- and NanoString nCounter data.

**Figure 1:**
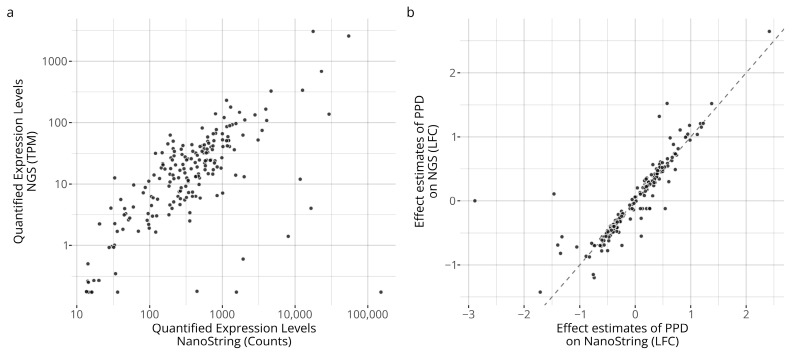
Comparison of quantified expression profiles between the platforms. Panel (**a**) shows average expression levels of the unstimulated control samples. Panel (**b**) compares the estimated effects induced by the positive control relative to the unstimulated control expressed in log2 fold changes (LFCs).

Following mapping and refinement, a total of 872 transcripts were used to reconstruct the 196 NanoString probe signals. The transcript identities and their mapping to the NanoString probes are described in Supplementary Table S2. A gene-by-gene comparison between the two platforms, based on the 24 paired samples, are displayed in Figure 1. As seen, the platform renders similar signals for majority of the measurements. Clear linear relationships can be seen between the platforms’ abundance estimates (Figure 1a) as well as a particularly strong correlation between the relative effect estimates (Figure 1b). However, it is also apparent that a few genes deviate more markedly from the overall associations. For the abundance estimate, these genes include *SNORA45*, five histone cluster genes (*HIST1H1C*, *HIST1H1E*, *HIST1H3G*, *HIST1H3J*, *HIST2H2AA3*), and *UFC1*. For the abundance estimates, the Pearson correlation coefficient was calculated to be 0.66 and the Spearman correlation coefficient was calculated to be 0.69. For the effect estimates, five histone cluster genes (*HIST1H1C*, *HIST1H1E*, *HIST1H3J*, *HIST2H2AA3*, *HIST2H2BF*) together with *ACER2* were identified as different between the platforms. The Pearson correlation coefficient was estimated to be 0.87, the Spearman correlation was estimated to be 0.95, and Lin’s concordance correlation coefficient was estimated to be 0.87.

**Figure 2:**
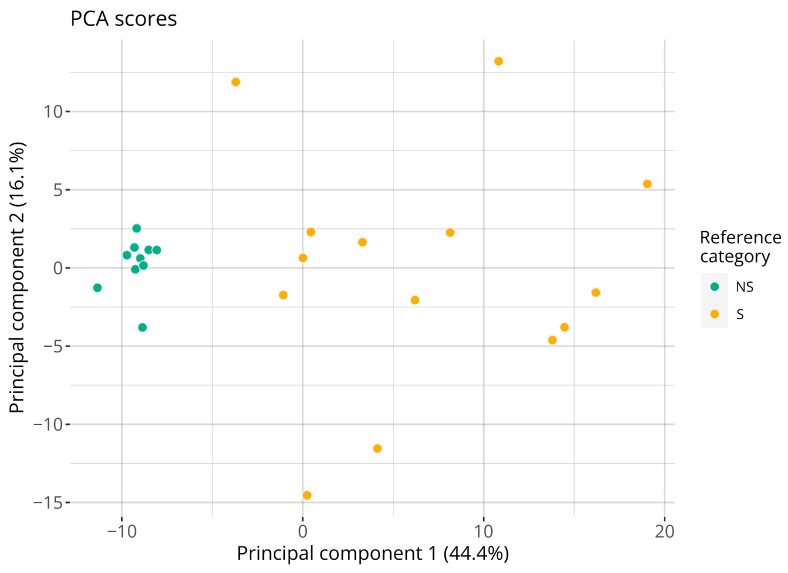
Visualization of the two first principal components of the RNA-seq dataset. Points are colored with respect to the consensus reference sensitizing category. Each point represents a distinct chemical. Abbreviations: NS: non-sensitizer; S: sensitizer.

The complete RNA-seq dataset, described using the 196 reconstructed probe signals, was visualized using PCA; see Figure 2. As seen, the reconstructed signals from RNA-seq data captures information relevant for discerning the sensitizers from the non-sensitizers. Indeed, the largest variance component allows for a complete separation between the sensitizing chemicals and the non-sensitizing ones.

### 3.2. Classifying Sensitizing Hazard with RNA-Seq Data

**Figure 3:**
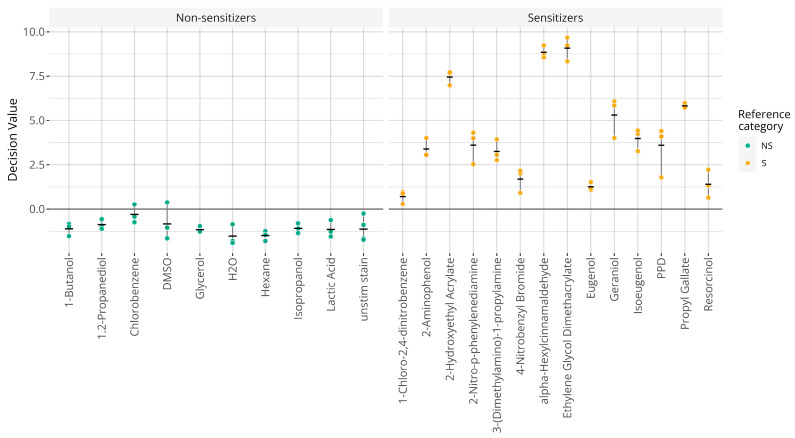
Graphical representation of GARDskin classifications. Points indicate predicted decision values for individual replicates and crossbars describe the mean decision values. Abbreviations: NS: non-sensitizer; S: sensitizer.

Hazard classifications based on RNA-seq data were then considered. The RNA-seq data for the 75 samples acquired for assessing predictive capacity comprised the reconstructed probe signals that were fed directly into the existing GARDskin analysis pipeline without making any modifications to it, i.e., all steps were performed as described in the GARDskin protocols with identical parameter values (as defined in the Supporting Document to the GARDskin assay [39]). Classifications and predicted decision values are visualized in Figure 3. As seen, all chemicals were correctly classified. Thus, on this dataset, the accuracy, sensitivity, and specificity were all 100%. Considering the decision values, the overall standard deviation between replicates was estimated to 0.64.

### 3.3. Assessment of Sequencing Depth

**Figure 4:**
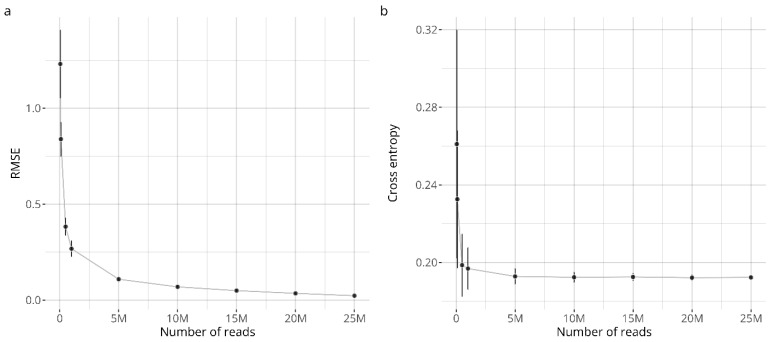
Classification performance of subsampled data. Panel (**a**) shows the root mean squared errors (RMSEs), comparing decision values of subsampled data with decision values from the complete dataset. Panel (**b**) shows the cross-entropy (log loss), comparing classifications with reference labels. The points describe the average value observed across the simulations and the error bars extend one standard deviation from the mean.

The sequencing depth required for making accurate GARDskin classifications was examined as an adjustable parameter in RNA-seq experiments. The effect of the sequencing depth was tested by subsampling the acquired RNA-seq data. Binomial subsampling was first used on the already quantified expression matrix, and the classification performance versus the number of quantified reads are visualized in Figure 4. As seen, the deterioration of the performance only becomes obvious at the lower sequencing depths, when ≤1 million counts were obtained.

From these observations, it was concluded that an average of 5 to 10 million reads per sample should be sufficient to maintain stable classification performance. The performance at both of these sequencing depths (5 M and 10 M) were verified by subsampling the reads already from the fastq files, and by rerunning the entire quantification and classification pipeline. The results were very similar to the data obtained by the binomial subsampling on the quantified reads. Indeed, the RMSE was estimated to be 0.153 and 0.116, and the cross-entropy was estimated to be 0.194 and 0.197 for the five- and ten-million count datasets, respectively.

## 4. Discussion

The development of non-animal methods (NAMs) for hazard assessment is continuously evolving, relying on technical advancement to make methods more capable of accurately and efficiently detecting and characterizing hazards. The assessment of skin sensitizers is one subfield where the progression of NAMs has led to the regulatory adoption of multiple individual assays [32,73,74], defined approaches (DAs), and integrated testing strategies (ITSs) [27]. The individual OECD-adopted assays currently include 10 test methods, with each described in a test guideline based on the understanding of the assay’s detection mechanism, in relation to the KEs of the AOP for skin sensitization. The GARDskin assay monitors dendritic cell activation by measuring changes in gene expression induced by test items, which include both neat chemicals and more complex formulations and solid materials [31,34,36,37].

The GARDskin biomarker signature (GPS) consists of 196 genes, which were identified and refined by exploratory analyses on whole transcriptome data [16]. The current testing protocol describes that gene level quantification should be performed using NanoString nCounter technologies. While it is an efficient platform for targeted acquisition, the ability to use gene expression levels from alternative quantification platforms could be beneficial. For example, technologies such as RNA-seq, which evolved rapidly during the last decades [75], have a wider user base and larger laboratory implementation compared with the NanoString nCounter platform. In addition, RNA-seq produces whole transcriptome data, offering interesting avenues for secondary analyses.

We have, therefore, explored the possibility of using gene expression data quantified with RNA-seq for GARDskin hazard classifications as an alternative technique to the currently supported NanoString nCounter platform. A total of 24 samples were acquired on both platforms as paired samples, and an addition set of 75 samples were acquired on the RNA-seq platform for assessment of predictive capacity. Using the paired samples, the acquired abundance estimates as well as the relative treatment effects were compared between the platforms. The abundance estimates showed a linear moderate or strong association between the platforms, as demonstrated in Figure 1a and as indicated by the Pearson correlation coefficient of 0.67. However, a few genes deviated more markedly from the observed relationship. The largest cross-platform differences, relative to the observed relationship, were seen for *SNORA45*, five histone cluster genes, and *UFC1*. The *SNORA45* target is a small nucleolar RNA (snoRNA), and the library preparation method of the RNA-seq experiment should explain the discrepancy, as the poly-A selection may not effectively capture them [76,77]. A similar explanation can likely also be attributed to the observed differences in the histone cluster genes, as they too lack the poly-A tail [78]. No definitive reason explaining the observed difference for the remaining *UFC1* gene has been identified.

While correlated abundance estimates would facilitate cross-platform predictions, potential differences could likely be mitigated if the relative effect differences between the platforms were similar. Figure 1b shows the estimated effects of the assay’s positive control, PPD, for both platforms. As seen, the estimates are strongly and linearly correlated. Furthermore, the estimates were very similar for most measurements, which can be observed in the graph as the datapoints are localized along the identity line (i.e., identical estimates for both platforms). However, again, a few genes showed a significantly different effect estimate. These corresponded to five histone cluster genes, which have already been discussed, and the *ACER2* gene. The *ACER2* gene is expressed barely above the limit of detection, and it is possible that the difference is linked to the assay’s dynamic range. It is to be noted that the effect size induced by PPD is estimated as relatively higher in the RNA-seq data compared with the NanoString platform. Taken together, and of great importance in relation to the context of the work, while small cross-platform discrepancies were observed, they have seemingly little to no effect on endpoint classifications, as derived from the GARDskin analysis pipeline, as further discussed below. The overall observations are also in line with previous comparisons between the two platforms, where strong agreements have been reported [53,79].

Having considered the performance of the individual genes, attention was given to the larger RNA-seq dataset and the chemicals acquired for assessment of predictive capacity. Figure 2 shows the PCA of the RNA-seq dataset based on the 196 target genes for the 24 treatments. As seen, the GARDskin prediction signature is capable of efficiently separating the skin-sensitizing chemicals from the non-sensitizing ones. Indeed, the first principal component achieves a complete separation between the classes. This strong separability further validates the biological relevance of the genes in the prediction signature and emphasizes that the variance is not dependent on specific quantification technologies.

The ability to generate accurate GARDskin classifications with RNA-seq data was then examined. Given the regulatory status of the assay, where the analysis pipeline and all of the fitted parameters have been explicitly defined, predictions and classifications were obtained by feeding the RNA-seq abundance estimates into the existing pipeline. Obtained predictions and classifications are displayed in Figure 3. As seen, all chemicals were correctly classified. This is also in complete concordance with historical results based on the standard protocols with NanoString quantification. Furthermore, the magnitudes of the decision values were very similar to what is routinely observed in the standard assay, and the variability between the replicates was also in line with expectations [27]. Thus, all observations based on the predictions indicate that the results were very similar to those of a typical testing scenario. No apparent deviations or biases could be identified based on the predicted decision values, which further corroborates the findings that quantification levels across the platforms were overall similar. It also indicates that the existing GARDskin analysis pipeline, which already includes steps designed to handle batch effects between a training set and a test set, is sufficiently robust to also handle differences identified for individual probes, as noted previously.

The final aspect that was considered concerned the required sequencing depth. The sequencing depth can be varied depending on requirements, and there is a natural trade-off between cost-effectiveness and data accuracy and reliability [80]. Based on subsampled data, GARDskin classifications could be effectively established from as little as five million quantified reads per sample.

## 5. Conclusions

GARDskin data quantified with the NanoString nCounter platform was compared with data from RNA-seq experiments. The results showed that the two platforms produced very similar estimates for majority of the genes. It was shown that gene expression levels quantified with RNA-seq could be used in the GARDskin assay to derive accurate hazard classifications that corresponded completely with the standard assay’s outcomes. The characteristics of the predictions were very similar to those observed in the standard assay, as currently described, suggesting that RNA-seq may be used as a gene expression quantification method to derive hazard classifications with the GARDskin assay. Indeed, given these results, the acquisition platforms could be considered interchangeably in a regulatory context for hazard assessment with GARDskin, following relevant approval procedures. Potential future research avenues could focus on the standardization of the RNAseq platform protocol and definition of quality criteria which would promote a more streamlined adoption in the GARDskin assay.

## Figures and Tables

**Table 1:** Description of assayed chemicals.

Chemical	CASRN	Input Concentration	LLNA MLLP (%)	LLNA Call	HDSG Call	Consensus Reference Call	N Replicates
4-Nitrobenzyl Bromide	100-11-8	2 µM	0.05	1	-	1	3
1-Chloro-2,4-dinitrobenzene	97-00-7	4 µM	0.054	1	1	1	3
p-Phenylenediamine (PPD)	106-50-3	75 µM	0.11	1	1	1	16 (12 + 4)
2-Nitro-p-phenylenediamine	5307-14-2	400 µM	0.4	1	-	1	3
2-Aminophenol	95-55-6	50 µM	0.45	1	-	1	3
Isoeugenol	97-54-1	300 µM	1.3	1	1	1	3
3-(Dimethylamino)-1-propylamine	109-55-7	500 µM	3.5	1	-	1	3
Resorcinol	108-46-3	500 µM	6.3	1	-	1	3
alpha-Hexylcinnamaldehyde	101-86-0	300 µM	10.8	1	-	1	3
Eugenol	97-53-0	500 µM	11.6	1	1	1	3
Geraniol	106-24-1	500 µM	16.1	1	1	1	3
Ethylene Glycol Dimethacrylate	97-90-5	500 µM	28	1	-	1	3
2-Hydroxyethyl Acrylate	818-61-1	100 µM	-	1	-	1	3
Propyl Gallate	121-79-9	100 µM	-	1	-	1	3
1-Butanol	71-36-3	500 µM	-	0	-	0	3
1.2-Propanediol	57-55-6	500 µM	-	0	0	0	3
Chlorobenzene	108-90-7	500 µM	-	0	-	0	3
DMSO (negative control)	67-68-5	0.1%	72	1	0	0	4
Glycerol	56-81-5	500 µM	-	0	-	0	3
H2O (negative control)	7732-18-5	0.1%	-	0	0	0	3
Hexane	110-54-3	500 µM	-	0	0	0	3
Isopropanol	67-63-0	500 µM	-	0	-	0	3
Lactic acid	50-21-5	500 µM	-	0	-	0	3
Unstimulated control	-	NA	-	0	0	0	16 (12 + 4)

LLNA MLLP (%) describes the median-like location parameter for EC3 values. LLNA Call describes the binary sensitization category based on LLNA data. HDGS Call describes the binary sensitization category based on human data. Consensus reference Call describes the binary consensus reference category based on both human and LLNA data. N Replicates describes the total number of replicate acquired for treatment in this study. P-phenylenediamine and unstimulated control have a total of 16 replicates. A total of twelve of these were used for the technical transfer based on paired samples, and four samples were used for assessment of predictive capacity. Abbreviations: CASRN: Chemical Abstract Service Registry Number; LLNA MLLP: local lymph node assay median location-like parameter; HDSG Call: binary sensitization call based on human data collated and interpreted by the human data sub-group [27].

## Data Availability

The datasets generated during and analyzed during the current study are available from the corresponding author on reasonable request.

## Supplementary Materials

The following supporting information can be downloaded at https://www.mdpi.com/article/10.3390/toxics14010009/s1; Table S1: programs, versions, and parameters used for RNA-seq data processing; Table S2: transcript IDs used to reconstruct the NanoString signals on the RNA-seq platform.

## Abbreviations

The following abbreviations are used in this manuscript:

AOP	Adverse Outcome Pathway
CASRN	Chemical Abstracts Service Registry Number
CPTC	Counts-per-total-counts
EC3	The effective concentration that induces a stimulation index of three in the LLNA assay
GPS	GARDskin Prediction Signature
HDSG	Human Data Sub-Group
KE	Key Event
LFC	Log Fold Change
LLNA	Local Lymph Node Assay
MLLP	Median-Like Location Parameter
NGS	Next Generation Sequencing
PCA	Principal Component Analysis
RMSE	Root Mean Squared Error
SVM	Support Vector Machine
TPM	Transcripts per Million
