# Supplementary material for: RNA-Seq Can Be Used to Quantify Gene Expression Levels for Use in the GARDskin Assay
Source: Toxics. 2025 Dec 20;14(1):9. doi: 10.3390/toxics14010009 (PMC12845768; doi:10.3390/toxics14010009)
Supplement: Supplementary file 1 [file toxics-14-00009-s001.zip › toxics-3923617-supplementary.pdf]

Supplementary materials

RNA-seq can be used to quantify gene expression levels for use in the GARDskin assay

Supplementary materials, Table *S*1. Programs, versions, and parameters used for RNA-seq data processing.

Program	Program version	Parameter	Value (if any)
bowtie2	2.5.4	-a	
bowtie2	2.5.4	--local	
fastp	0.23.4	-f	1
fastp	0.23.4	--adapter_sequence	CTGTCTCTTATACACATC
fastp	0.23.4	--adapter_sequence_r2	CTGTCTCTTATACACATCT
salmon	1.10.0	-l	A
salmon	1.10.0	-validateMappings	
salmon	1.10.0	-softclip	
fq subsample	0.12.0	-s	[random seed]
fq subsample	0.12.0	--probability	[values to render 5M & 10M reads on average]

Supplementary materials, Table *S*2. Transcript IDs used to reconstruct the NanoString signals on the RNA-seq platform.

NanoString ID	Transcript ID
**AAMDC**	ENST00000393427.7
**AAMDC**	ENST00000532481.5
**AAMDC**	ENST00000526164.5
**AAMDC**	ENST00000526415.5
**AAMDC**	ENST00000527134.5
**AAMDC**	ENST00000304716.12
**AAMDC**	ENST00000533193.5
**ABAT**	ENST00000565016.5
**ABAT**	ENST00000562115.1
**ABAT**	ENST00000569156.5
**ABAT**	ENST00000268251.13
**ABAT**	ENST00000396600.6
**ABAT**	ENST00000565671.1
**ABHD5**	ENST00000454293.2
**ABHD5**	ENST00000643520.1

**ABHD5**	ENST00000644371.2
**ABHD5**	ENST00000643140.1
**ABHD5**	ENST00000649763.1
**ABHD5**	ENST00000643500.1
**ABHD5**	ENST00000646378.1
**ABHD5**	ENST00000642351.1
**ACER2**	ENST00000340967.3
**ACLY**	ENST00000353196.5
**ACLY**	ENST00000352035.7
**ACLY**	ENST00000590151.5
**ACTR10**	ENST00000545307.6
**ACTR10**	ENST00000553907.1
**ACTR10**	ENST00000254286.9
**ACTR10**	ENST00000554402.6
**ACTR10**	ENST00000555229.5
**ACTR10**	ENST00000555337.5
**ADAM20**	ENST00000256389.5
**ALDH18A1**	ENST00000371224.7
**ALDH18A1**	ENST00000371221.3
**ALDH1B1**	ENST00000377698.4
**ALKBH6**	ENST00000378875.8
**ALKBH6**	ENST00000490483.5
**ALKBH6**	ENST00000490986.5
**ALKBH6**	ENST00000461668.5
**ALKBH6**	ENST00000471323.1
**ALKBH6**	ENST00000486389.5
**ALKBH6**	ENST00000252984.11
**ANAPC1 LOC730268**	ENST00000778692.1
**ANAPC1 LOC730268**	ENST00000341068.8
**ANAPC5**	ENST00000541887.5
**ANAPC5**	ENST00000441917.6
**ANAPC5**	ENST00000534976.5
**ANAPC5**	ENST00000535482.1
**ANAPC5**	ENST00000261819.8
**ANAPC5**	ENST00000545218.5
**ANKRA2**	ENST00000296785.8
**ANKRA2**	ENST00000515804.1
**ARFGAP3**	ENST00000263245.10
**ARHGAP9**	ENST00000546200.5
**ARHGAP9**	ENST00000430041.6
**ASB7**	ENST00000332783.12
**ASB7**	ENST00000343276.4

**ATP6V0D1**	ENST00000563305.1
**ATP6V0D1**	ENST00000540149.5
**ATP6V0D1**	ENST00000426604.7
**ATP6V0D1**	ENST00000568620.5
**ATP6V0D1**	ENST00000290949.8
**ATP6V0D1**	ENST00000567694.5
**ATP6V0E1**	ENST00000519911.5
**ATP6V0E1**	ENST00000519374.6
**ATP6V0E1**	ENST00000265093.4
**ATP6V1H**	ENST00000520188.5
**ATP6V1H**	ENST00000355221.7
**ATP6V1H**	ENST00000524164.5
**ATP6V1H**	ENST00000359530.7
**BCL7A**	ENST00000261822.5
**BCL7A**	ENST00000538010.5
**BIN2**	ENST00000604560.6
**BIN2**	ENST00000605039.5
**BIN2**	ENST00000544402.5
**BIN2**	ENST00000605819.1
**BIN2**	ENST00000615107.6
**BIN2**	ENST00000452142.7
**BIN2**	ENST00000504996.1
**BIN2**	ENST00000503900.1
**BIN2**	ENST00000811219.1
**BLMH**	ENST00000261714.11
**C12orf57**	ENST00000542222.1
**C12orf57**	ENST00000544681.1
**C12orf57**	ENST00000538392.1
**C12orf57**	ENST00000607421.3
**C12orf57**	ENST00000229281.6
**C12orf57**	ENST00000537087.5
**C19orf54**	ENST00000470681.5
**C19orf54**	ENST00000378313.7
**C19orf54**	ENST00000598352.1
**C1orf174**	ENST00000680054.1
**C1orf174**	ENST00000361605.4
**C20orf24**	ENST00000494506.1
**C20orf24**	ENST00000558530.1
**C20orf24**	ENST00000492721.5
**C20orf24**	ENST00000344795.8
**C20orf24**	ENST00000373852.9
**C20orf24**	ENST00000342422.3

**C9orf89**	ENST00000490488.5
**C9orf89**	ENST00000466929.5
**C9orf89**	ENST00000466409.1
**C9orf89**	ENST00000375464.7
**C9orf89**	ENST00000498243.1
**C9orf89**	ENST00000475574.1
**CARM1**	ENST00000327064.9
**CARM1**	ENST00000586221.5
**CARM1**	ENST00000344150.8
**CD33**	ENST00000600557.1
**CD33**	ENST00000262262.5
**CD33**	ENST00000421133.6
**CD33**	ENST00000601785.5
**CD86**	ENST00000330540.7
**CD86**	ENST00000478741.1
**CD93**	ENST00000246006.5
**COX20**	ENST00000391839.6
**COX20**	ENST00000411948.7
**COX20**	ENST00000498262.1
**COX20**	ENST00000464757.1
**COX20**	ENST00000452636.1
**COX7A2L**	ENST00000234301.3
**CRHBP**	ENST00000274368.9
**CRHBP**	ENST00000503763.1
**CSGALNACT2**	ENST00000374466.4
**CYP51A1**	ENST00000003100.13
**CYP51A1**	ENST00000691309.1
**CYP51A1**	ENST00000719830.1
**CYP51A1**	ENST00000419457.1
**CYP51A1**	ENST00000492267.1
**DDRGK1**	ENST00000354488.8
**DDRGK1**	ENST00000496781.1
**DDX19A**	ENST00000302243.12
**DDX19A**	ENST00000562140.2
**DDX21**	ENST00000687162.1
**DDX21**	ENST00000685106.1
**DDX21**	ENST00000354185.9
**DDX21**	ENST00000620315.2
**DDX21**	ENST00000684824.1
**DDX21**	ENST00000686528.1
**DHCR24**	ENST00000649769.1
**DHCR24**	ENST00000436604.2

**DHCR24**	ENST00000648728.1
**DHCR24**	ENST00000535035.6
**DHCR24**	ENST00000371269.9
**DHCR7**	ENST00000355527.8
**DHCR7**	ENST00000407721.6
**DHCR7**	ENST00000526780.6
**DHCR7**	ENST00000683714.1
**DHCR7**	ENST00000682708.1
**DHX33**	ENST00000572490.1
**DHX33**	ENST00000433302.7
**DHX33**	ENST00000225296.8
**DNAJB4**	ENST00000370763.6
**DNAJB4**	ENST00000487931.1
**DNAJB9**	ENST00000491582.1
**DNAJB9**	ENST00000249356.4
**DNAJC5**	ENST00000470551.1
**DNAJC5**	ENST00000360864.9
**DNAJC9**	ENST00000372950.6
**DRAM2**	ENST00000496430.6
**DRAM2**	ENST00000539140.6
**DRAM2**	ENST00000484310.6
**DTD1**	ENST00000377452.4
**DTD1**	ENST00000647441.1
**EDEM2**	ENST00000374492.8
**EMC7**	ENST00000256545.9
**EMC7**	ENST00000532113.1
**EVI2B**	ENST00000577894.1
**EVI2B**	ENST00000330927.5
**FAM212B**	ENST00000444059.2
**FAM212B**	ENST00000357260.6
**FAM212B**	ENST00000534365.1
**FAM86A**	ENST00000458008.8
**FAM86A**	ENST00000587133.1
**FAM86A**	ENST00000427587.9
**FAM86A**	ENST00000477247.6
**FAM86A**	ENST00000484945.6
**FAM86A**	ENST00000735504.1
**FAM86A**	ENST00000690856.1
**FAM86A**	ENST00000735447.1
**FAM86A**	ENST00000734393.1
**FAM86A**	ENST00000734391.1
**FAM86A**	ENST00000512092.6

**FAM86A**	ENST00000693359.1
**FAM86A**	ENST00000735469.1
**FAM86A**	ENST00000736774.1
**FAM86A**	ENST00000736782.1
**FAM86A**	ENST00000735678.1
**FAM86A**	ENST00000735671.1
**FAM86A**	ENST00000735674.1
**FAM86A**	ENST00000735628.1
**FAM86A**	ENST00000763997.1
**FAM86A**	ENST00000763992.1
**FAM86A**	ENST00000522601.5
**FAM86A**	ENST00000834657.1
**FAM86A**	ENST00000774996.1
**FAM86A**	ENST00000775015.1
**FAM86A**	ENST00000775007.1
**FAM86A**	ENST00000775004.1
**FAM86A**	ENST00000775000.1
**FAM86A**	ENST00000688253.2
**FAM86A**	ENST00000533513.1
**FAM86A**	ENST00000737141.1
**FAM86A**	ENST00000737148.1
**FAM86A**	ENST00000737135.1
**FAM86A**	ENST00000737164.1
**FAM86A**	ENST00000737131.1
**FAM86A**	ENST00000737171.1
**FAM86A**	ENST00000737126.1
**FAM86A**	ENST00000737132.1
**FAM86A**	ENST00000737162.1
**FAS**	ENST00000357339.7
**FAS**	ENST00000696744.1
**FAS**	ENST00000696774.1
**FAS**	ENST00000460510.6
**FAS**	ENST00000697036.1
**FAS**	ENST00000487314.1
**FAS**	ENST00000690268.1
**FAS**	ENST00000355740.8
**FAS**	ENST00000640140.2
**FAS**	ENST00000696996.1
**FAS**	ENST00000355279.2
**FAS**	ENST00000697037.1
**FAS**	ENST00000696777.1
**FAS**	ENST00000612663.6

**FAS**	ENST00000697095.1
**FAS**	ENST00000696741.1
**FASN**	ENST00000634990.1
**FASN**	ENST00000306749.4
**FBXO10 RP11.613M10.8**	ENST00000544475.5
**FBXO10 RP11.613M10.8**	ENST00000276960.7
**FBXO10 RP11.613M10.8**	ENST00000432825.7
**FDPSP2 FDPSP7**	ENST00000764296.1
**FDXR**	ENST00000293195.10
**FDXR**	ENST00000420580.6
**FDXR**	ENST00000581530.5
**FDXR**	ENST00000577509.5
**FDXR**	ENST00000583917.5
**FDXR**	ENST00000442102.6
**FDXR**	ENST00000582944.5
**FOXO4**	ENST00000374259.8
**FTH1P2**	ENST00000533138.1
**FTH1P2**	ENST00000450676.1
**FTH1P2**	ENST00000394290.3
**FTH1P2**	ENST00000273550.12
**FTH1P2**	ENST00000401830.3
**FTH1P2**	ENST00000430907.2
**FTH1P2**	ENST00000425761.1
**FTH1P2**	ENST00000620041.5
**FTH1P2**	ENST00000498161.1
**FTH1P2**	ENST00000435241.1
**FTH1P2**	ENST00000832861.1
**FTH1P2**	ENST00000406984.2
**FTH1P2**	ENST00000492727.1
**FTH1P2**	ENST00000480365.1
**FTH1P2**	ENST00000437933.1
**FTH1P2**	ENST00000557194.1
**FTH1P2**	ENST00000512179.1
**FTH1P2**	ENST00000534719.1
**FTH1P2**	ENST00000449131.6
**FTH1P2**	ENST00000529548.1
**FTH1P5**	ENST00000491005.6
**FTH1P5**	ENST00000471408.5
**FTH1P5**	ENST00000286186.11
**FTH1P5**	ENST00000374747.9
**FTH1P5**	ENST00000593682.1
**FUCA2**	ENST00000415586.5

**FUCA2**	ENST00000002165.11
**FUCA2**	ENST00000619849.4
**FUCA2**	ENST00000622321.1
**FUCA2**	ENST00000610068.5
**FUCA2**	ENST00000591189.5
**FUCA2**	ENST00000593045.5
**FUCA2**	ENST00000590703.5
**FUCA2**	ENST00000593175.1
**FUCA2**	ENST00000438118.6
**FUCA2**	ENST00000612298.1
**GAS2L3**	ENST00000539410.2
**GAS2L3**	ENST00000547754.6
**GAS2L3**	ENST00000266754.9
**GDAP2**	ENST00000369442.3
**GDAP2**	ENST00000369443.10
**GDF11**	ENST00000257868.10
**GLRX**	ENST00000237858.11
**GLRX**	ENST00000379979.8
**GLRX**	ENST00000508780.5
**GLRX**	ENST00000555594.1
**GLRX**	ENST00000470810.1
**GLRX**	ENST00000423261.1
**GNL3L**	ENST00000360845.3
**GNPNAT1**	ENST00000216410.8
**GNPNAT1**	ENST00000554230.5
**GNPNAT1**	ENST00000554421.1
**GNPNAT1**	ENST00000555689.1
**GNPNAT1**	ENST00000650397.1
**GSR**	ENST00000643653.1
**GSR**	ENST00000221130.11
**GSR**	ENST00000643525.1
**GSR**	ENST00000537535.5
**GTF3C2**	ENST00000264720.8
**GTF3C2**	ENST00000457098.5
**GTF3C2**	ENST00000359541.6
**HBP1**	ENST00000468410.5
**HBP1**	ENST00000498408.1
**HBP1**	ENST00000222574.9
**HBP1**	ENST00000461963.1
**HBP1**	ENST00000483809.1
**HBP1**	ENST00000463790.1
**HIST1H1C**	ENST00000343677.4

**HIST1H1C**	ENST00000244534.7
**HIST1H1E**	ENST00000304218.6
**HIST1H2AE**	ENST00000303910.5
**HIST1H2AE**	ENST00000621411.3
**HIST1H2AE**	ENST00000608318.3
**HIST1H2BE HIST1H2BC HIST1H2BI HIST1H2BF HIST1H2BG**	ENST00000614097.3
**HIST1H2BE HIST1H2BC HIST1H2BI HIST1H2BF HIST1H2BG**	ENST00000634910.1
**HIST1H2BE HIST1H2BC HIST1H2BI HIST1H2BF HIST1H2BG**	ENST00000707189.1
**HIST1H2BE HIST1H2BC HIST1H2BI HIST1H2BF HIST1H2BG**	ENST00000396984.2
**HIST1H2BE HIST1H2BC HIST1H2BI HIST1H2BF HIST1H2BG**	ENST00000707188.1
**HIST1H2BE HIST1H2BC HIST1H2BI HIST1H2BF HIST1H2BG**	ENST00000314332.5
**HIST1H2BE HIST1H2BC HIST1H2BI HIST1H2BF HIST1H2BG**	ENST00000377733.4
**HIST1H2BE HIST1H2BC HIST1H2BI HIST1H2BF HIST1H2BG**	ENST00000377401.4
**HIST1H2BE HIST1H2BC HIST1H2BI HIST1H2BF HIST1H2BG**	ENST00000274764.5
**HIST1H3G**	ENST00000635641.1
**HIST1H3G**	ENST00000356476.3
**HIST1H3G**	ENST00000618052.2
**HIST1H3J HIST1H3F HIST1H3B HIST1H3H HIST1H3G HIST1H3I HIST1H3E HIST1H3C HIST1H3D HIST1H3A**	ENST00000359303.4
**HIST1H4A HIST2H4B HIST4H4 HIST2H4A HIST1H4L HIST1H4E HIST1H4B HIST1H4H HIST1H4C HIST1H4J H**	ENST00000355057.3
**HIST1H4A HIST2H4B HIST4H4 HIST2H4A HIST1H4L HIST1H4E HIST1H4B HIST1H4H HIST1H4C HIST1H4J H**	ENST00000611927.2
**HIST2H2AA3 HIST2H2AA4_x1**	ENST00000331380.4
**HIST2H2AA3 HIST2H2AA4_x1**	ENST00000715903.1
**HIST2H2AA3 HIST2H2AA4_x2**	ENST00000607355.3
**HIST2H2AA3 HIST2H2AA4_x2**	ENST00000331380.4
**HIST2H2AA3 HIST2H2AA4_x2**	ENST00000530167.2
**HIST2H2BF**	ENST00000609879.2
**HMGB3**	ENST00000325307.12
**HMGCR**	ENST00000343975.9
**HMGCR**	ENST00000681567.1
**HMGCR**	ENST00000680160.1
**HMGCR**	ENST00000287936.9

**HMGCR**	ENST00000681410.1
**HMGCR**	ENST00000679456.1
**HMGCR**	ENST00000681271.1
**HMGCR**	ENST00000680940.1
**HMGCS1**	ENST00000507004.1
**HMGCS1**	ENST00000511774.1
**HMGCS1**	ENST00000325110.11
**HMGCS1**	ENST00000433297.2
**HMGCS1**	ENST00000400943.3
**HMOX1**	ENST00000679074.1
**HMOX1**	ENST00000677931.1
**HMOX1**	ENST00000481190.2
**HMOX1**	ENST00000216117.9
**HNRNPL**	ENST00000647557.2
**HNRNPL**	ENST00000221419.10
**HNRNPL**	ENST00000388749.7
**HNRNPL**	ENST00000600873.5
**HNRNPL**	ENST00000594769.5
**IRS2**	ENST00000375856.5
**ISCU**	ENST00000431221.6
**ISCU**	ENST00000545932.5
**ISCU**	ENST00000311893.14
**ISCU**	ENST00000538193.1
**ISCU**	ENST00000539580.5
**ISG20L2**	ENST00000368219.2
**ISG20L2**	ENST00000472824.2
**ISG20L2**	ENST00000313146.11
**KCNE3**	ENST00000759310.1
**KCNE3**	ENST00000310128.9
**KCNE3**	ENST00000530510.1
**KGFLP1 KGFLP2 RP11.204M4.2 RP11.111F5.5**	ENST00000754626.1
**KGFLP1 KGFLP2 RP11.204M4.2 RP11.111F5.5**	ENST00000809287.1
**KGFLP1 KGFLP2 RP11.204M4.2 RP11.111F5.5**	ENST00000711658.2
**KGFLP1 KGFLP2 RP11.204M4.2 RP11.111F5.5**	ENST00000717397.1
**KGFLP1 KGFLP2 RP11.204M4.2 RP11.111F5.5**	ENST00000717403.1
**KGFLP1 KGFLP2 RP11.204M4.2 RP11.111F5.5**	ENST00000509595.2
**KGFLP1 KGFLP2 RP11.204M4.2 RP11.111F5.5**	ENST00000809275.1
**KGFLP1 KGFLP2 RP11.204M4.2 RP11.111F5.5**	ENST00000809235.1
**KGFLP1 KGFLP2 RP11.204M4.2 RP11.111F5.5**	ENST00000809232.1
**KGFLP1 KGFLP2 RP11.204M4.2 RP11.111F5.5**	ENST00000560979.1
**KGFLP1 KGFLP2 RP11.204M4.2 RP11.111F5.5**	ENST00000809284.1
**KGFLP1 KGFLP2 RP11.204M4.2 RP11.111F5.5**	ENST00000809288.1

**KIAA0226L**	ENST00000417405.2
**KIAA0226L**	ENST00000676307.1
**KIAA0226L**	ENST00000378787.7
**LOC100499405 RP11.118B22.3 RP11.118B22.2**	ENST00000838866.1
**LOC100499405 RP11.118B22.3 RP11.118B22.2**	ENST00000838873.1
**LOC100499405 RP11.118B22.3 RP11.118B22.2**	ENST00000647751.1
**LOC100996496 SFPQ**	ENST00000357214.6
**LOC100996496 SFPQ**	ENST00000696553.1
**LOC101060521 POLR3E**	ENST00000615879.4
**LOC101060521 POLR3E**	ENST00000299853.10
**LPAR1**	ENST00000374431.7
**LPAR1**	ENST00000683809.1
**LPAR1**	ENST00000374430.6
**LPAR1**	ENST00000358883.8
**LRPPRC**	ENST00000260665.12
**LY96**	ENST00000518893.1
**LY96**	ENST00000284818.7
**MAP2K1**	ENST00000691576.1
**MAP2K1**	ENST00000689951.1
**MAP2K1**	ENST00000307102.10
**MAP2K1**	ENST00000692683.1
**MAP2K1**	ENST00000685172.1
**MAP2K1**	ENST00000685763.1
**MAP2K1**	ENST00000519737.1
**MAPK13**	ENST00000373766.9
**MAPK13**	ENST00000211287.9
**MAPK13**	ENST00000373759.1
**METTL2A**	ENST00000311506.10
**METTL2A**	ENST00000333483.14
**METTL2A**	ENST00000262432.13
**METTL2A**	ENST00000482555.5
**MGST3 LOC100505828**	ENST00000627653.1
**MGST3 LOC100505828**	ENST00000367888.8
**MGST3 LOC100505828**	ENST00000367883.3
**MGST3 LOC100505828**	ENST00000495447.5
**MGST3 LOC100505828**	ENST00000367889.8
**MGST3 LOC100505828**	ENST00000367885.5
**MGST3 LOC100505828**	ENST00000488688.1
**MGST3 LOC100505828**	ENST00000609263.1
**MIR4271 C3orf62**	ENST00000343010.8
**MRPL30 C2orf15**	ENST00000338148.8
**MRPL4**	ENST00000393733.6

**MRPL4**	ENST00000591054.5
**MRPL4**	ENST00000590702.1
**MRPL4**	ENST00000307422.9
**MRPL4**	ENST00000253099.11
**MRPS17**	ENST00000443449.1
**MRPS17**	ENST00000285298.9
**MRPS17**	ENST00000426595.1
**MRPS17**	ENST00000772451.1
**MRPS17**	ENST00000772450.1
**MRPS17**	ENST00000772452.1
**MRPS17**	ENST00000511924.1
**MRPS17**	ENST00000465453.1
**MRPS17**	ENST00000622571.1
**MSANTD2**	ENST00000239614.8
**MSANTD2**	ENST00000650095.1
**MSANTD2**	ENST00000374979.8
**MSANTD2**	ENST00000674284.1
**MSANTD2**	ENST00000526629.1
**MTR**	ENST00000366577.10
**MTR**	ENST00000680454.1
**MTR**	ENST00000674797.2
**MYBBP1A**	ENST00000254718.9
**MYBBP1A**	ENST00000573116.5
**NBR1**	ENST00000341165.10
**NBR1**	ENST00000590996.6
**NIP7**	ENST00000567202.5
**NIP7**	ENST00000563364.3
**NIP7**	ENST00000254941.6
**NIP7**	ENST00000254940.10
**NLRP12**	ENST00000391773.8
**NLRP12**	ENST00000391772.1
**NLRP12**	ENST00000492915.1
**NLRP12**	ENST00000345770.9
**NLRP12**	ENST00000391775.7
**NLRP12**	ENST00000324134.11
**NOL6**	ENST00000353159.6
**NOL6**	ENST00000297990.9
**NQO1**	ENST00000320623.10
**NQO1**	ENST00000379047.7
**NQO1**	ENST00000379046.6
**NQO1**	ENST00000564043.1
**NQO1**	ENST00000561500.5

**NRBP1**	ENST00000233557.7
**NRBP1**	ENST00000460499.5
**NRBP1**	ENST00000379852.8
**NUBPL**	ENST00000281081.12
**NUBPL**	ENST00000552489.5
**NUBPL**	ENST00000547839.5
**NUBPL**	ENST00000551314.1
**NUDT14**	ENST00000550912.1
**NUDT14**	ENST00000392568.7
**NUFIP1**	ENST00000379161.5
**NUFIP1**	ENST00000402902.1
**NUP153**	ENST00000262077.3
**OR5B21**	ENST00000360374.3
**OSER1 C20orf111**	ENST00000255174.3
**OSER1 C20orf111**	ENST00000372970.6
**PASK**	ENST00000544142.5
**PASK**	ENST00000493544.1
**PASK**	ENST00000358649.8
**PASK**	ENST00000405260.5
**PASK**	ENST00000403638.7
**PASK**	ENST00000234040.9
**PAWR**	ENST00000328827.9
**PAWR**	ENST00000551712.1
**PDAP1**	ENST00000350498.8
**PDAP1**	ENST00000392530.2
**PDE1B**	ENST00000548855.5
**PDE1B**	ENST00000243052.8
**PDE1B**	ENST00000542335.5
**PDE1B**	ENST00000611899.4
**PDE1B**	ENST00000550620.1
**PDE1B**	ENST00000538346.5
**PFAS**	ENST00000314666.11
**PHLDA3**	ENST00000367311.5
**PHLDA3**	ENST00000485436.1
**PIK3AP1**	ENST00000371110.6
**PIK3AP1**	ENST00000339364.10
**PINK1**	ENST00000492302.1
**PINK1**	ENST00000321556.5
**PMM2**	ENST00000565896.5
**PMM2**	ENST00000564030.5
**PMM2**	ENST00000268261.9
**PMM2**	ENST00000682393.1

**PMM2**	ENST00000683274.1
**PMM2**	ENST00000562448.1
**PMM2**	ENST00000569958.5
**PNO1**	ENST00000263657.7
**PNO1**	ENST00000604029.1
**PNO1**	ENST00000756729.1
**PNO1**	ENST00000756730.1
**POLR2E**	ENST00000215587.11
**POLR2E**	ENST00000589737.5
**POLR2E**	ENST00000586817.5
**POLR2E**	ENST00000590060.5
**POLR2E**	ENST00000612655.4
**POLR2E**	ENST00000615234.5
**POLR2E**	ENST00000591709.1
**PPM1D**	ENST00000692386.1
**PPM1D**	ENST00000693196.1
**PPM1D**	ENST00000305921.8
**PPM1D**	ENST00000685212.1
**PREX1**	ENST00000371941.4
**PSTPIP1**	ENST00000379595.7
**PSTPIP1**	ENST00000559295.5
**PSTPIP1**	ENST00000558012.6
**RAB33B**	ENST00000652268.1
**RAB33B**	ENST00000305626.6
**RENBP**	ENST00000471056.5
**RENBP**	ENST00000423624.5
**RENBP**	ENST00000393700.8
**RENBP**	ENST00000369997.7
**RENBP**	ENST00000475904.1
**RFC2**	ENST00000470266.5
**RFC2**	ENST00000621097.4
**RFC2**	ENST00000352131.7
**RFC2**	ENST00000055077.8
**RFC2**	ENST00000494019.5
**RFC2**	ENST00000415505.1
**RNASEH1**	ENST00000315212.4
**RNASEH1**	ENST00000436842.5
**RNASEH1**	ENST00000658393.1
**RNF146**	ENST00000608991.5
**RNF146**	ENST00000356799.6
**RNF146**	ENST00000368314.6
**RNF146**	ENST00000610153.1

**RNF24**	ENST00000336095.10
**RNF24**	ENST00000545616.2
**RNF24**	ENST00000432261.6
**RNF24**	ENST00000358395.11
**RNF26**	ENST00000311413.5
**RP11.267J23.4**	ENST00000397627.3
**RP11.267J23.4**	ENST00000341369.11
**RP11.267J23.4**	ENST00000481928.1
**RP11.267J23.4**	ENST00000466801.5
**RP11.267J23.4**	ENST00000412128.1
**RP11.267J23.4**	ENST00000448874.5
**RP11.267J23.4**	ENST00000839378.1
**RP11.267J23.4**	ENST00000838862.1
**RP11.267J23.4**	ENST00000843685.1
**RP11.267J23.4**	ENST00000409115.8
**RP11.267J23.4**	ENST00000427111.5
**RP11.267J23.4**	ENST00000426188.2
**RP11.267J23.4**	ENST00000410064.5
**RP11.267J23.4**	ENST00000607242.1
**RP11.267J23.4**	ENST00000468027.5
**RP11.267J23.4**	ENST00000556323.1
**RP11.267J23.4**	ENST00000635020.1
**RP11.267J23.4**	ENST00000409683.5
**RP11.267J23.4**	ENST00000602494.1
**RP11.267J23.4**	ENST00000447474.1
**RPF2P1**	ENST00000424137.2
**RPF2P1**	ENST00000441448.7
**RPF2P1**	ENST00000607388.1
**RPSA SNORA62 SNORA6**	ENST00000458478.6
**RPSA SNORA62 SNORA6**	ENST00000697731.1
**RPSA SNORA62 SNORA6**	ENST00000301821.11
**RPSA SNORA62 SNORA6**	ENST00000444512.2
**RPSA SNORA62 SNORA6**	ENST00000477325.1
**RPSA SNORA62 SNORA6**	ENST00000697728.1
**RPSA SNORA62 SNORA6**	ENST00000697729.1
**RPSA SNORA62 SNORA6**	ENST00000488229.1
**RPSA SNORA62 SNORA6**	ENST00000442699.1
**RPSA SNORA62 SNORA6**	ENST00000458337.5
**RPSA SNORA62 SNORA6**	ENST00000433498.1
**RPSA SNORA62 SNORA6**	ENST00000437336.1
**RPSA SNORA62 SNORA6**	ENST00000418207.1
**RPSA SNORA62 SNORA6**	ENST00000744127.1

**RPSA SNORA62 SNORA6**	ENST00000744129.1
**RPSA SNORA62 SNORA6**	ENST00000429844.2
**RPSA SNORA62 SNORA6**	ENST00000848559.1
**RPSA SNORA62 SNORA6**	ENST00000483479.1
**RPSA SNORA62 SNORA6**	ENST00000641751.1
**RPSA SNORA62 SNORA6**	ENST00000401872.1
**RPSA SNORA62 SNORA6**	ENST00000445182.2
**RPSA SNORA62 SNORA6**	ENST00000465940.1
**RPSA SNORA62 SNORA6**	ENST00000485258.1
**RPSA SNORA62 SNORA6**	ENST00000477347.1
**RPSA SNORA62 SNORA6**	ENST00000453374.2
**RPSA SNORA62 SNORA6**	ENST00000467439.1
**RPSA SNORA62 SNORA6**	ENST00000744128.1
**RPSA SNORA62 SNORA6**	ENST00000456555.1
**RPSA SNORA62 SNORA6**	ENST00000445739.2
**RPSA SNORA62 SNORA6**	ENST00000412013.1
**RPSA SNORA62 SNORA6**	ENST00000415189.1
**RPSA SNORA62 SNORA6**	ENST00000414293.2
**RPSA SNORA62 SNORA6**	ENST00000447583.2
**RPSA SNORA62 SNORA6**	ENST00000590033.1
**RPSA SNORA62 SNORA6**	ENST00000806298.1
**RPSA SNORA62 SNORA6**	ENST00000406712.1
**RPSA SNORA62 SNORA6**	ENST00000806299.1
**RPSA SNORA62 SNORA6**	ENST00000493929.1
**RPSA SNORA62 SNORA6**	ENST00000489520.2
**RPSA SNORA62 SNORA6**	ENST00000446095.1
**RPSA SNORA62 SNORA6**	ENST00000441455.1
**RPSA SNORA62 SNORA6**	ENST00000461543.1
**RPSA SNORA62 SNORA6**	ENST00000584105.1
**RPSA SNORA62 SNORA6**	ENST00000432681.1
**RPSA SNORA62 SNORA6**	ENST00000625020.1
**RPSA SNORA62 SNORA6**	ENST00000497462.2
**RPSA SNORA62 SNORA6**	ENST00000475346.1
**RPSA SNORA62 SNORA6**	ENST00000760598.1
**RPSA SNORA62 SNORA6**	ENST00000760600.1
**RPSA SNORA62 SNORA6**	ENST00000662434.1
**RPSA SNORA62 SNORA6**	ENST00000760599.1
**RPSA SNORA62 SNORA6**	ENST00000760602.1
**RPSA SNORA62 SNORA6**	ENST00000421323.1
**RPSA SNORA62 SNORA6**	ENST00000425966.6
**RPSA SNORA62 SNORA6**	ENST00000635702.1
**RPSA SNORA62 SNORA6**	ENST00000648951.1

**RPSA SNORA62 SNORA6**	ENST00000771209.1
**RPSA SNORA62 SNORA6**	ENST00000657394.1
**RPSA SNORA62 SNORA6**	ENST00000848451.1
**RPUSD2**	ENST00000315616.12
**RPUSD2**	ENST00000616318.1
**RRP12**	ENST00000622320.4
**RRP12**	ENST00000370992.9
**RRP12**	ENST00000315563.10
**RXRA**	ENST00000481739.2
**RXRA**	ENST00000356384.4
**SCARB2**	ENST00000638603.1
**SCARB2**	ENST00000639715.1
**SCARB2**	ENST00000264896.8
**SCARB2**	ENST00000640640.1
**SCARB2**	ENST00000640341.1
**SCARB2**	ENST00000638295.1
**SCARB2**	ENST00000639145.1
**SERBP1**	ENST00000361219.11
**SERBP1**	ENST00000370994.8
**SLC25A32**	ENST00000649416.1
**SLC25A32**	ENST00000523866.1
**SLC25A32**	ENST00000707124.1
**SLC25A32**	ENST00000521645.5
**SLC35B3**	ENST00000644923.2
**SLC35B3**	ENST00000648867.1
**SLC35B3**	ENST00000648987.1
**SLC35B3**	ENST00000649788.1
**SLC35B3**	ENST00000379660.4
**SLC35B3**	ENST00000710437.1
**SLC37A4**	ENST00000330775.9
**SLC37A4**	ENST00000357590.9
**SLC37A4**	ENST00000538950.5
**SLC37A4**	ENST00000697850.1
**SLC37A4**	ENST00000526275.5
**SLC37A4**	ENST00000638925.1
**SLC37A4**	ENST00000697847.1
**SLC37A4**	ENST00000697845.1
**SLC37A4**	ENST00000529510.6
**SLC37A4**	ENST00000697848.1
**SLC37A4**	ENST00000525102.5
**SLC37A4**	ENST00000638186.1
**SLC37A4**	ENST00000525039.5

**SLC37A4**	ENST00000532888.6
**SLC37A4**	ENST00000526626.6
**SLC37A4**	ENST00000650539.1
**SLC37A4**	ENST00000527992.5
**SLC37A4**	ENST00000697846.1
**SLC37A4**	ENST00000697849.1
**SLC5A6**	ENST00000428518.5
**SLC5A6**	ENST00000426119.5
**SLC5A6**	ENST00000401463.5
**SLC5A6**	ENST00000445802.5
**SLC5A6**	ENST00000310574.8
**SLC5A6**	ENST00000432106.5
**SLC5A6**	ENST00000442731.1
**SMPD4**	ENST00000439886.5
**SMPD4**	ENST00000351288.10
**SMPD4**	ENST00000433118.5
**SMPD4**	ENST00000482171.5
**SMPD4**	ENST00000431183.6
**SMPD4**	ENST00000409031.5
**SMPD4**	ENST00000850260.1
**SNHG1 SNORD26 SNORD22 SNORD25 SNORD27 SNORD28 SNORD31 SNORD29**	ENST00000741088.1
**SNHG1 SNORD26 SNORD22 SNORD25 SNORD27 SNORD28 SNORD31 SNORD29**	ENST00000742703.1
**SNHG1 SNORD26 SNORD22 SNORD25 SNORD27 SNORD28 SNORD31 SNORD29**	ENST00000742554.1
**SNHG1 SNORD26 SNORD22 SNORD25 SNORD27 SNORD28 SNORD31 SNORD29**	ENST00000658540.2
**SNHG1 SNORD26 SNORD22 SNORD25 SNORD27 SNORD28 SNORD31 SNORD29**	ENST00000545440.6
**SNHG1 SNORD26 SNORD22 SNORD25 SNORD27 SNORD28 SNORD31 SNORD29**	ENST00000538654.6
**SNHG1 SNORD26 SNORD22 SNORD25 SNORD27 SNORD28 SNORD31 SNORD29**	ENST00000742573.1
**SNHG1 SNORD26 SNORD22 SNORD25 SNORD27 SNORD28 SNORD31 SNORD29**	ENST00000742474.1
**SNHG1 SNORD26 SNORD22 SNORD25 SNORD27 SNORD28 SNORD31 SNORD29**	ENST00000537925.5
**SNHG1 SNORD26 SNORD22 SNORD25 SNORD27 SNORD28 SNORD31 SNORD29**	ENST00000742552.1
**SNHG1 SNORD26 SNORD22 SNORD25 SNORD27 SNORD28 SNORD31 SNORD29**	ENST00000742576.1

**SNHG1 SNORD26 SNORD22 SNORD25 SNORD27 SNORD28 SNORD31 SNORD29**	ENST00000668048.2
**SNHG1 SNORD26 SNORD22 SNORD25 SNORD27 SNORD28 SNORD31 SNORD29**	ENST00000664307.2
**SNHG1 SNORD26 SNORD22 SNORD25 SNORD27 SNORD28 SNORD31 SNORD29**	ENST00000742502.1
**SNHG1 SNORD26 SNORD22 SNORD25 SNORD27 SNORD28 SNORD31 SNORD29**	ENST00000670323.2
**SNHG1 SNORD26 SNORD22 SNORD25 SNORD27 SNORD28 SNORD31 SNORD29**	ENST00000661936.1
**SNHG1 SNORD26 SNORD22 SNORD25 SNORD27 SNORD28 SNORD31 SNORD29**	ENST00000742564.1
**SNHG1 SNORD26 SNORD22 SNORD25 SNORD27 SNORD28 SNORD31 SNORD29**	ENST00000742607.1
**SNHG1 SNORD26 SNORD22 SNORD25 SNORD27 SNORD28 SNORD31 SNORD29**	ENST00000665512.2
**SNHG1 SNORD26 SNORD22 SNORD25 SNORD27 SNORD28 SNORD31 SNORD29**	ENST00000540725.7
**SNHG1 SNORD26 SNORD22 SNORD25 SNORD27 SNORD28 SNORD31 SNORD29**	ENST00000742637.1
**SNHG1 SNORD26 SNORD22 SNORD25 SNORD27 SNORD28 SNORD31 SNORD29**	ENST00000742538.1
**SNHG1 SNORD26 SNORD22 SNORD25 SNORD27 SNORD28 SNORD31 SNORD29**	ENST00000742659.1
**SNHG1 SNORD26 SNORD22 SNORD25 SNORD27 SNORD28 SNORD31 SNORD29**	ENST00000742467.1
**SNHG1 SNORD26 SNORD22 SNORD25 SNORD27 SNORD28 SNORD31 SNORD29**	ENST00000742712.1
**SNHG1 SNORD26 SNORD22 SNORD25 SNORD27 SNORD28 SNORD31 SNORD29**	ENST00000742567.1
**SNHG1 SNORD26 SNORD22 SNORD25 SNORD27 SNORD28 SNORD31 SNORD29**	ENST00000742609.1
**SNHG1 SNORD26 SNORD22 SNORD25 SNORD27 SNORD28 SNORD31 SNORD29**	ENST00000742679.1
**SNHG1 SNORD26 SNORD22 SNORD25 SNORD27 SNORD28 SNORD31 SNORD29**	ENST00000742582.1
**SNHG1 SNORD26 SNORD22 SNORD25 SNORD27 SNORD28 SNORD31 SNORD29**	ENST00000742608.1
**SNHG1 SNORD26 SNORD22 SNORD25 SNORD27 SNORD28 SNORD31 SNORD29**	ENST00000365607.2
**SNHG1 SNORD26 SNORD22 SNORD25 SNORD27 SNORD28 SNORD31 SNORD29**	ENST00000662400.1

**SNHG1 SNORD26 SNORD22 SNORD25 SNORD27 SNORD28 SNORD31 SNORD29**	ENST00000742559.1
**SNHG12 SNORA16A SNORA61 SNORA44**	ENST00000475441.7
**SNHG12 SNORA16A SNORA61 SNORA44**	ENST00000648251.2
**SNHG12 SNORA16A SNORA61 SNORA44**	ENST00000461832.3
**SNHG12 SNORA16A SNORA61 SNORA44**	ENST00000775065.1
**SNHG12 SNORA16A SNORA61 SNORA44**	ENST00000775061.1
**SNHG12 SNORA16A SNORA61 SNORA44**	ENST00000481220.6
**SNHG12 SNORA16A SNORA61 SNORA44**	ENST00000461448.6
**SNHG12 SNORA16A SNORA61 SNORA44**	ENST00000464612.5
**SNHG12 SNORA16A SNORA61 SNORA44**	ENST00000775062.1
**SNORA45**	ENST00000408166.1
**SNX27**	ENST00000368838.2
**SNX27**	ENST00000368841.7
**SNX27**	ENST00000642349.1
**SNX27**	ENST00000643937.1
**SNX27**	ENST00000644970.1
**SNX27**	ENST00000368843.8
**SPRY2**	ENST00000377102.5
**SPRY2**	ENST00000377104.4
**SQLE**	ENST00000265896.10
**SREBF2**	ENST00000361204.9
**SRP72P2**	ENST00000344851.4
**SRP72P2**	ENST00000646579.1
**SRP72P2**	ENST00000510663.6
**SRP72P2**	ENST00000642900.1
**ST3GAL6**	ENST00000483910.6
**ST3GAL6**	ENST00000394162.5
**ST3GAL6**	ENST00000486249.5
**ST3GAL6**	ENST00000613264.5
**ST3GAL6**	ENST00000469105.5
**ST3GAL6**	ENST00000491912.1
**ST3GAL6**	ENST00000474595.1
**STK17B**	ENST00000714420.1
**STK17B**	ENST00000263955.9
**STK17B**	ENST00000714417.1
**STK17B**	ENST00000420683.2
**STK17B**	ENST00000409228.5
**STK17B**	ENST00000449152.2
**STK17B**	ENST00000714419.1
**STK17B**	ENST00000714421.1
**TAPT1**	ENST00000405303.7

**TAPT1**	ENST00000505603.5
**TAS2R5**	ENST00000247883.5
**TBCEL**	ENST00000683345.1
**TBCEL**	ENST00000284259.11
**TBCEL**	ENST00000531148.5
**TBCEL**	ENST00000533169.1
**TBCEL**	ENST00000422003.6
**TCTN2**	ENST00000680574.1
**TCTN2**	ENST00000303372.7
**TCTN2**	ENST00000680500.1
**TCTN2**	ENST00000426174.6
**TCTN2**	ENST00000679504.1
**TLR6**	ENST00000381950.2
**TLR6**	ENST00000508254.6
**TLR9**	ENST00000494383.1
**TLR9**	ENST00000360658.3
**TLR9**	ENST00000478201.1
**TMEM150B**	ENST00000586609.5
**TMEM150B**	ENST00000326652.9
**TMEM150B**	ENST00000585918.5
**TMEM150B**	ENST00000592731.5
**TMEM55A**	ENST00000520709.5
**TMEM55A**	ENST00000285419.8
**TMEM55A**	ENST00000518359.5
**TMEM59**	ENST00000452421.5
**TMEM59**	ENST00000371337.3
**TMEM59**	ENST00000234831.10
**TMEM97**	ENST00000582384.1
**TMEM97**	ENST00000336687.6
**TMEM97**	ENST00000226230.8
**TMEM97**	ENST00000517841.1
**TOMM34**	ENST00000372813.4
**TOMM40**	ENST00000252487.9
**TOMM40**	ENST00000426677.7
**TOMM40**	ENST00000592434.5
**TOMM40**	ENST00000405636.6
**TP53I3**	ENST00000407482.5
**TP53I3**	ENST00000417886.1
**TP53I3**	ENST00000238721.9
**TP53I3**	ENST00000335934.8
**TP53I3**	ENST00000413037.1
**TP53INP1**	ENST00000448464.6

**TP53INP1**	ENST00000342697.5
**TWF2**	ENST00000678882.1
**TWF2**	ENST00000678352.1
**TWF2**	ENST00000676988.1
**TWF2**	ENST00000499914.2
**TWF2**	ENST00000305533.10
**TWF2**	ENST00000677127.1
**TWF2**	ENST00000678330.1
**TWF2**	ENST00000679180.1
**TWF2**	ENST00000679296.1
**TWF2**	ENST00000676800.1
**TXNRD1**	ENST00000526691.5
**TXNRD1**	ENST00000503506.6
**TXNRD1**	ENST00000527688.5
**UFC1**	ENST00000637249.1
**USP10**	ENST00000563892.5
**USP10**	ENST00000569038.5
**USP10**	ENST00000219473.12
**USP10**	ENST00000540269.6
**USP10**	ENST00000563048.5
**USP10**	ENST00000563433.1
**USP10**	ENST00000548656.2
**USP10**	ENST00000547785.2
**VAMP3**	ENST00000054666.11
**VARS_x1**	ENST00000375663.8
**VARS_x2**	ENST00000375663.8
**VPS37A**	ENST00000520140.5
**VPS37A**	ENST00000425020.6
**VPS37A**	ENST00000521829.5
**VPS37A**	ENST00000324849.9
**VPS37A**	ENST00000520639.1
**X7966223**	ENST00000365601.1
**X8133549**	ENST00000451013.7
**X8140170**	ENST00000619775.1
**X8140170**	ENST00000620662.1
**X8145244**	ENST00000397703.6
**X8145244**	ENST00000520607.1
**YBX1P1**	ENST00000445822.1
**YBX1P1**	ENST00000321358.12
**YBX1P1**	ENST00000489612.1
**YBX1P1**	ENST00000442962.2
**YBX1P1**	ENST00000467957.1

**YBX1P1**	ENST00000468366.1
**ZNF211**	ENST00000535785.1
**ZNF211**	ENST00000254182.11
**ZNF211**	ENST00000407202.6
**ZNF211**	ENST00000540556.5
**ZNF211**	ENST00000391703.3
**ZNF211**	ENST00000347302.7
**ZNF211**	ENST00000240731.5
**ZNF211**	ENST00000299871.9
**ZNF223**	ENST00000434772.8
**ZNF223**	ENST00000591793.1
**ZNF561**	ENST00000424629.5
**ZNF561**	ENST00000302851.8
**ZNF561**	ENST00000326044.9
**ZNF561**	ENST00000293648.8
**ZNF561**	ENST00000443819.5
**ZNF79**	ENST00000612342.4
**ZNF79**	ENST00000617266.2
**ZNF79**	ENST00000342483.5
